# Correlative Study of Aggression in Adult Patients With Mood Disorder Admitted in a Tertiary Hospital From 2015 to 2017: A Retrospective Study

**DOI:** 10.7759/cureus.106100

**Published:** 2026-03-30

**Authors:** Jeanne Leigh L Arboleda, Carmina Bernardo, Elaine Angela Leynes, Ormalyn Bayting, Robin A Dantes, Vichka Sison, Ofelia Abayan, Sophia Benosa, Lorraine Freyja Linsangan

**Affiliations:** 1 Psychiatry, Makati Medical Center, Makati, PHL

**Keywords:** aggression, anxiety, bipolar disorder, major depressive disorder, mood disorders, suicidal behavior, violence

## Abstract

Introduction

Aggressive acts demonstrated by patients suffering from mood disorders can vary from irritability to homicidal or suicidal behavior. It has been reported that patients with affective disorder, specifically bipolar disorder, most recent episode mixed, have a higher odds of aggressive behavior compared to those with schizophrenia. The investigators were intrigued to explore how suicidality correlated with other factors and determinants of mood disorder by classifying it as an act of aggression toward oneself, giving credence to the psychopathological notion of suicidality.

Methods

The study was designed as a retrospective cross-sectional analytic investigation that will utilize a database previously gathered. The existing database has 705 data sets and has already been processed to be non-identifiable data. Upon processing, there were 373 data sets gathered and organized through the use of a data collection tool. The data the investigators gathered were the demographics and clinical determinants, as they have been hypothesized to have a relationship. Statistical analysis was done using Student's t-test for continuous variables and chi-square test or Fisher's exact test for categorical variables. Simple logistic regression was done to determine the variables that probably have significant association with aggression.

Results

The study showed a predominance of patients without aggression (266 from a total of 373). With the P-value noted to be significant if <0.20, only civil status was noted to be significant (p=0.004998) in the demographic variables. In the clinical determinants (p<0.20), the mood state during admission (p=2.798e^-10^), mood symptoms during admission (p=1.127e^-12^), the type of aggression (p=2.2e^-16^), and the total number of mood symptoms, depressed (p=3.462e^-10^) and manic (p=3.445e^-11^), were significant. Admitting diagnosis (p=0.004498), discharge diagnosis (p=0.001499), and comorbid psychiatric disorder (p=0.04998) were also noted to be significant in determining the presence or absence of aggression. Individuals in manic, mixed, or anxious states can expect their outcomes to be 5-6 times greater than those who are depressed. These values were also noted to be significant (p<0.20) with manic (p=1.59435e^-09^), mixed (p=2.60205e^-04^), and anxious (p=2.880880e^-07^).

Conclusions

It can be safely concluded that the factors with significant association to the absence and presence of aggression (civil status, mood state during admission, patients' mood symptoms at the time of admission, the type of aggression, the total number of symptoms for depression and mania, admitting diagnosis, discharge diagnosis, and comorbid psychiatric disorders) are also known considerations for assessing the probability of aggression, either toward others or of suicidal nature. The recent mood state was shown to have a positive association in terms of predicting the probability of aggression, especially manic, anxious, and mixed states. One of the primary purposes of this inquiry was to examine whether there is a change in terms of predictors of aggression if the concept of aggression includes any violence committed toward oneself.

## Introduction

Aggressive acts demonstrated by patients suffering from mood disorders can vary from irritability to homicidal or suicidal behavior [1]. Even the criterion A of major depressive disorder in the Diagnostic and Statistical Manual, fifth edition, cites that one of the symptoms that can be seen in a major depressive episode is suicidality [2]. It has been observed and demonstrated that emotions have a causative relation to aggressive behavior, whether it be anger, jealousy, or shame [3].

It has been reported that patients with affective disorder, specifically bipolar disorder, most recent episode mixed, have a higher odds of aggressive behavior compared to those with schizophrenia [4]. Another study reports that patients diagnosed with bipolar I disorder presenting with psychotic features state that a recent suicidal attempt has a strong association with aggression [1]. There is even a distinction made between those who are classified as suicidal ideators who have a lower risk for aggression, specifically violence, and suicidal attempters in cases of major depressive disorder [5].

A more direct link was demonstrated in this study, and it shows that unipolar depression correlated negatively with aggression. It was seen to be frequently present in patients having mixed features. The presence of aggression, regardless of form or typology, was associated with the higher severity of manic and depressive episodes. Also, patients with aggression have higher rates of psychiatric comorbidity, such as substance abuse or borderline personality disorder [6].

Aggression

A concise statement defining aggression can be seen in Kaplan and Sadock's comprehensive review of psychiatry. There, it was described as an overt behavior with a threat or an act that can possibly or actually cause damage in any form. It can be a reaction to a perceived intent of harm toward an individual or an unprovoked action shown by an individual. It might be an act that is socially acceptable or vilified in the public eye. Every society can have a difference in the standard of what is acceptable [7].

Aggression is classified in multiple typologies as it can be seen from multiple dimensions. It can be about the social acceptability (sanctioned versus non-sanctioned), in terms of the arousal of the individual committing the act (hypoarousal versus hyperarousal), and whether a provoked action or simply by impulse (proactive versus reactive) [7]. The direction wherein aggression is expressed can also be considered a dimension of aggression, as some literature has coined the term intra-aggression (directed inward) and extra-aggression (directed outward) [8]. Also, the form it takes (verbal versus nonverbal) has been one of the aspects studied in the ongoing literature of aggression [3].

With most of these factors in mind, it would be curious to ask how aggression manifests in patients within the context of mood disorders. Does aggression have a causative relationship with the mood state of the patient, are there other factors to consider in predicting aggression within the context of the mood changes, or are there certain elements that influence the aggression while in a particular mood state? These are some of the inquiries that the investigators have elucidated in this study. The investigators were intrigued to explore how suicidality correlated with other factors and determinants of mood disorder by classifying it as an act of aggression toward oneself, giving credence to the psychopathological notion of suicidality. Also, it is necessary to investigate how the factors considered as risks for suicide are observed in the context of our local setting. In this particular undertaking, the prevalence of aggression and its associated risk factors in adult patients with mood disorders admitted to a tertiary hospital were considered variables. In addition, the study focuses specifically on (1) describing aggressive behavior seen in adult patients with mood disorders, (2) correlating the presence of aggression to their respective mood disorders with regard to the demographics and clinical determinants, and (3) determining how the mood state or episode is associated with the aggressive behavior.

## Materials and methods

The study was designed as a retrospective cross-sectional analytic investigation that will utilize a database. The existing database has 705 data sets and has already been processed to be non-identifiable data upon gathering the necessary factors that are relevant to the study [9]. Upon processing, there were 375 data sets gathered and organized through the use of a data collection tool (Appendices). All the patient identifiers were removed, and the data sets were identified by the assigned control numbers for privacy purposes.

Inclusion and exclusion criteria

The inquiry was focused on data sets that had a diagnosis of major depressive disorder or bipolar disorder, accounting for their variants and coexisting psychiatric or medical conditions, from January 2015 to December 2017. The diagnostic criteria used to qualify the diagnosis of the cases in the sample were based on the American Psychiatric Association Diagnostic and Statistical Manual, fifth edition [2].

The data sets that had an admitting and discharge diagnosis of schizophrenia and other schizophrenia spectrum and other psychotic disorders; anxiety disorders; obsessive-compulsive and related disorders; trauma- and/or stressor-related disorders; dissociative disorders; somatic symptom and related disorders; elimination disorders; sleep-wake disorders; eating disorders; sexual dysfunction; disruptive, impulse-control, and conduct disorders; neurocognitive disorders; personality disorders; and paraphilic disorders as established by the American Psychiatric Association Diagnostic and Statistical Manual, fifth edition, were excluded.

Sampling

The sample size was derived by consulting a statistician, wherein it was computed using an online software (OpenEpi version 3, www.OpenEpi.com). The hypothesized percentage frequency of outcome was 58.7%, which is based on the prevalence estimate of any aggressive outbursts among patients with major depressive disorder [9]. The margin of error used is 5%. At 95% confidence level, the result showed that the minimum required sample size is 373. As stated above, we have 705 data sets available with the consideration of the exclusion and inclusion criteria; this will be further decreased with the age parameter, but since the sample size computed is nearly half, there is a distinctly high probability that the needed sample size was fulfilled. This study employed a random sampling method in which all patients with mood disorders were numbered from 1 to N, where N is the total number of patients. Random numbers will be generated using Microsoft Excel (Microsoft Corp., Redmond, WA), and it will be checked if the patient will satisfy the inclusion and exclusion criteria until the required sample size is completed.

Variables

**Figure 1:**
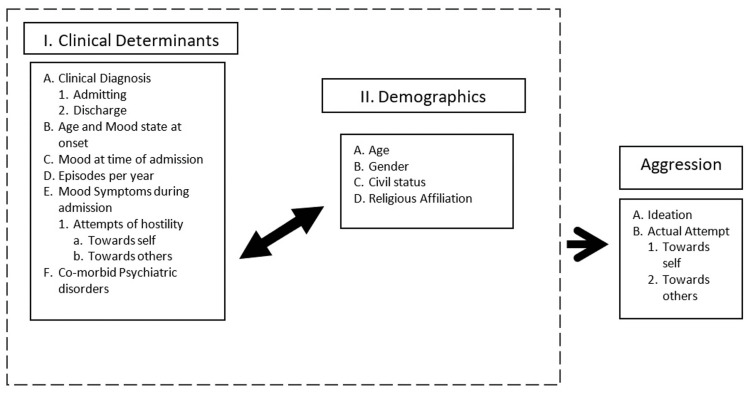
Conceptual Framework

The data the investigators gathered were the demographics and clinical determinants, as they have been hypothesized to have a relationship, as established from the studies in the literature. As shown in Figure 1, demographics pertain to the gender, age, civil or marital status, religious affiliation, and employment at the time of admission. Clinical determinants include details in the history, specifically admitting and final diagnoses during the most current hospitalization, mood state and the time of admission, and the number of episodes per year. In addition to this information, it is in the interest of the investigators to know the following details during the admission: patients' mood symptoms at the time of admission, any symptoms of aggression, any comorbid psychiatric or medical conditions, history of suicide attempt or self-harm and number of attempts, and mood state at the time of suicide. Statistically analyzing both the clinical determinants and demographics determines how the aggression presents itself. The data were gathered with the use of the data collection tool in the Appendices.

Statistical analysis

Descriptive statistics, such as the mean, was used to present data on the demographic and clinical profiles of the included patients. Frequency and percentages were used to present categorical data. Differences in the characteristics between patients with and without aggression will be compared using Student's t-test if continuous variables and chi-square test or Fisher's exact test if categorical variables.

Simple logistic regression was done to determine the variables that probably have significant association with aggression. P-values of less than 0.20 were considered to be significant. All variables that met the criteria were included in multiple logistic regression, followed by stepwise regression using backward selection to determine which variables had significant predictors of aggression among patients with mood disorders at the 0.05 level of significance.

Data security

The biggest concern in this undertaking would be handling the data. For identification purposes, the data sets were given a unique three-digit controlled number for the duration of the study. After the collection of sufficient numbers as specified by the sample size, the data sets were organized with the data collection tool as a guide. Upon finishing the collection and organization, the data were analyzed with the help of the statistician. The interpretation and conclusion were done by the primary investigator alone. All the gathered data were stored in a working laptop for three months after the study to countercheck any other discrepancies should there be any. After the specified duration, the said data sets in the working laptop of the primary investigator will be permanently removed. Any printed materials will be subject to shredding to avoid any confidentiality breach.

## Results

The demographics table (Table 1) showed a predominance of patients without aggression (266 from a total of 373). With the P-value noted to be significant if <0.20, only civil status was noted to be significant (p=0.004998), using the chi-square test. With a median age of 28, only data from adults were to be considered in the study. For gender, there is at least a 2:1 ratio among female-to-male data samples. Looking closely, you can still see the predominance in numbers for those who did not have aggression, especially in the female population (n=183). Going to the other side of the table, under the classification of those with aggression, there is a switch between the ratios, with the male data sample increased compared to the female data sets. Civil status, on the other hand, showed some consistency with the distribution on both classifications, with singles having no aggression at the top at 198. Religious affiliation was notably predominated by the Catholic denomination (n=300). On further classification of aggression, it showed that there are more non-Catholics with aggression (n=87) compared to Catholics with aggression (n=20). In employment, like civil status, it showed consistency, comparing the samples of both with aggression and without aggression, the latter being more in numbers.

**Table 1:** Demographics and Comparison of Variables With and Without the Presence of Aggression Data are presented as median and frequency (%). P-values<0.20 are significant ^c^Chi-square test ^m^Mann-Whitney U test

Demographic	Total (n=373)	Aggression		P-value
		Without (n=266)	With (n=107)	
Age	28 (20-44)	26 (20-39.8)	31 (21-48.5)	0.0489^m^
Gender				0.1712^c^
Male	125 (33.5)	83 (31.20)	65 (60.75)	-
Female	248 (66.5)	183 (68.80)	42 (39.25)	-
Civil status				0.004998^c^
Single	261 (70.0)	198 (74.44)	63 (58.88)	-
Married	90 (24.1)	51 (19.17)	39 (36.45)	-
Separated	15 (4.02)	11 (4.14)	4 (3.74)	-
Widowed	7 (1.88)	6 (2.26)	1 (0.93)	-
Religious affiliation				0.8987^c^
Catholic	300 (80.4)	213 (80.08)	20 (18.69)	-
Non-Catholic	73 (19.6)	53 (19.92)	87 (81.31)	-
Employment				0.508^c^
Student	118 (31.6)	89 (33.46)	29 (27.10)	-
Retired	25 (6.70)	17 (6.39)	8 (7.48)	-
Employed	147 (39.4)	105 (39.47)	42 (39.25)	-
Unemployed	70 (18.7)	48 (18.06)	22 (20.56)	-
Unknown	13 (3.49)	7 (2.63)	6 (5.61)	-

The clinical determinants shown in Table 2 with a notable significant difference with P-value at <0.20 are the following: the mood state during admission (p=2.798e^-10^), mood symptoms during admission (p=1.127e^-12^), the type of aggression (p=2.2e^-16^), and the total number of mood symptoms, depressed (p=3.462e^-10^) and manic (p=3.445e^-11^). The mood state during admission showed the predominance of a depressed state (n=207) and without aggression (n=178). In the column with aggression, it showed that there are more individuals with the manic state than the other mood states (n=39). Also, the euthymic state was noted to have no sample data classified as aggression. In the patients' mood symptoms at the time of admission, the number of depressed mood symptoms was still increased in the overall count (n=203). In addition, in the aggression part of Table 2, manic symptoms were noted to have the lead in terms of numbers (n=54). In the type of aggression, there was a noted difference in classifications. Some data samples showed that there are patients with a classification of aggression toward oneself, but they were noted to have no history of aggression. This simply means that data samples show episodes of self-directed harming (suicidal behavior, such as cutting and overdosing) but no episode of aggression toward others. Also, there was a noted prevalence of those classified under such a condition (n=112). Another classification of aggression that was analyzed was the suicidal attempts. For these, it can be seen that the majority of the samples had no availability of data (n=236) and the next in numbers is the yes category (n=135). In the number of mood episodes, there was no notable difference between the categories having 1-2 episodes per year and having one episode per year. In terms of the number of symptoms, the data sample showed that there is an inclination for those with depressive symptoms to have no history of aggression, while the data samples classified with manic symptoms were noted to have a history of aggression.

**Table 2:** Clinical Determinants and Comparison of Variables With and Without the Presence of Aggression Data are presented as median and frequency (%). P-values<0.20 are significant ^c^Chi-square test ^m^Mann-Whitney U test NA: not available

Clinical Determinants	Total (n=373)	Aggression		P-value
		Without n=266	With n=107	
Mood state during admission				2.798e^-10c^
Depressed	207 (55.5)	178 (66.92)	29 (27.10)	-
Manic	79 (21.2)	40 (15.04)	39 (36.45)	-
Mixed	23 (6.17)	12 (4.51)	11 (10.28)	-
Anxious	57 (15.3)	30 (11.28)	27 (25.23)	-
Unspecified	4 (1.07)	3 (1.13)	1 (0.93)	-
Euthymic	3 (0.804)	3 (1.13)	0	-
Patients' mood symptoms at the time of admission				1.127e^-12c^
Depressed	203 (54.4)	175 (65.79)	28 (26.17)	-
Manic	100 (26.8)	46 (17.29)	54 (50.47)	-
Mixed	70 (18.8)	45 (16.92)	25 (23.36)	-
Type of aggression				2.2e^-16c^
Toward oneself	125 (33.5)	112 (99.12)	13 (12.62)	-
Toward others	71 (19.0)	1 (.88)	70 (67.96)	-
Both	20 (5.36)	0	20 (19.42)	-
NA	157 (42.1)	153 (57.52)	4 (3.74)	-
History of suicide attempt or self-harm				0.1477^c^
Yes	135 (36.2)	105 (39.47)	30 (28.04)	-
Unknown	1 (0.268)	1	0	-
None	1 (0.268)	1	0	-
Not available	236 (63.3)	159 (59.77)	77 (71.96)	-
Number of mood episodes per year	1 (1-2)	1 (1-2)	1 (1-2)	0.8078^m^
Total number of symptoms	6 (5-8)	6 (5-8)	6 (5-8)	0.1953^m^
Total number of symptoms (depression)	5 (0-6)	5 (3-7)	0 (0-5)	3.462e^-10m^
Total number of symptoms (mania/hypomania)	0 (0-4)	0 (0-3)	4 (0-5.5)	3.445e^-11m^
Number of attempt(s)	2 (1-3)	2 (1-3)	1 (1-2.75)	0.1251^m^

Table 3 shows the admitting diagnosis in the data sample that was summarized. The majority of it was still under the diagnosis of major depressive disorder (n=95). Overall, it showed a significant difference at 0.004498 under Fisher's exact test (p<0.20).

**Table 3:** Admitting Diagnosis Data are presented as median and frequency (%). P-values<0.20 are significant ^f^Fisher's exact test

Admitting Diagnosis	Total (n=373)	Aggression		P-value
		Without (n=266)	With (n=107)	
Adjustment disorder	8 (2.14)	6 (0.023)	2 (0.019)	0.004498^f^
Adjustment disorder with depressed mood	4 (1.07)	3 (0.011)	1 (0.009)	-
Adjustment disorder with depressed mood and anxiety	1 (0.27)	1 (0.004)	0 (0)	-
Anxiety disorder, not otherwise specified	2 (0.54)	2 (0.008)	0 (0)	-
Bipolar 1 disorder	7 (1.88)	4 (0.015)	3 (0.028)	-
Bipolar 1 disorder with psychotic features	2 (0.54)	1 (0.004)	1 (0.009)	-
Bipolar 1 disorder, most recent episode depressed	18 (4.83)	12 (0.045)	6 (0.056)	-
Bipolar 1 disorder, most recent episode depressed, in anxious distress	1 (0.27)	1 (0.004)	0 (0)	-
Bipolar 1 disorder, most recent episode depressed, with mixed and psychotic features	1 (0.27)	1 (0.004)	0 (0)	-
Bipolar 1 disorder, most recent episode depressed, with psychotic features	3 (0.8)	2 (0.008)	1 (0.009)	-
Bipolar 1 disorder, most recent episode manic	55 (14.75)	25 (0.094)	30 (0.28)	-
Bipolar 1 disorder, most recent episode manic, with mixed features	1 (0.27)	1 (0.004)	0 (0)	-
Bipolar 1 disorder, most recent episode manic, with psychotic features	18 (4.83)	9 (0.034)	9 (0.084)	-
Bipolar 1 disorder, most recent episode mixed	6 (1.61)	5 (0.019)	1 (0.009)	-
Bipolar 1 disorder, most recent episode mixed, with psychotic features	2 (0.54)	1 (0.004)	1 (0.009)	-
Bipolar 1 disorder, most recent episode mixed, with psychotic features, in anxious distress	1 (0.27)	0 (0)	1 (0.009)	-
Bipolar 1 disorder, with mixed features, in anxious distress	2 (0.54)	2 (0.008)	0 (0)	-
Bipolar 2 disorder	4 (1.07)	3 (0.011)	1 (0.009)	-
Bipolar 2 disorder, in anxious distress	1 (0.27)	1 (0.004)	0 (0)	-
Bipolar 2 disorder, most recent episode depressed	8 (2.14)	7 (0.026)	1 (0.009)	-
Bipolar 2 disorder, most recent episode depressed, with mixed features	1 (0.27)	1 (0.004)	0 (0)	-
Bipolar 2 disorder, most recent episode hypomanic, with psychotic features	1 (0.27)	1 (0.004)	0 (0)	-
Bipolar 2 disorder, most recent episode mixed	2 (0.54)	1 (0.004)	1 (0.009)	-
Bipolar 2 disorder, most recent episode hypomanic, with mixed features	1 (0.27)	1 (0.004)	0 (0)	-
Bipolar disorder	31 (8.31)	20 (0.075)	11 (0.103)	-
Bipolar disorder with mixed and psychotic features	1 (0.27)	1 (0.004)	0 (0)	-
Bipolar disorder with psychotic features	5 (1.34)	3 (0.011)	2 (0.019)	-
Bipolar disorder, most recent episode depressed	6 (1.61)	5 (0.019)	1 (0.009)	-
Bipolar disorder, most recent episode mixed	1 (0.27)	0 (0)	1 (0.009)	-
Cannabis-induced mood disorder	1 (0.27)	0 (0)	1 (0.009)	-
Chronic insomnia	2 (0.54)	2 (0.008)	0 (0)	-
Delusional disorder	1 (0.27)	0 (0)	1 (0.009)	-
Depressive disorder	1 (0.27)	1 (0.004)	0 (0)	-
Disruptive mood dysregulation disorder	1 (0.27)	0 (0)	1 (0.009)	-
Generalized anxiety disorder	1 (0.27)	1 (0.004)	0 (0)	-
Major depressive disorder	95 (25.47)	80 (0.301)	15 (0.14)	-
Major depressive disorder with mixed features, in anxious distress	1 (0.27)	1 (0.004)	0 (0)	-
Major depressive disorder with psychotic features	28 (7.51)	20 (0.075)	8 (0.075)	-
Major depressive disorder with psychotic features, in anxious distress	1 (0.27)	1 (0.004)	0 (0)	-
Major depressive disorder, in anxious distress	22 (5.9)	20 (0.075)	2 (0.019)	-
Major depressive disorder, recurrent, with mixed and psychotic features	1 (0.27)	1 (0.004)	0 (0)	-
Mood disorder	11 (2.95)	9 (0.034)	2 (0.019)	-
Mood disorder with psychotic features	2 (0.54)	2 (0.008)	0 (0)	-
Mood disorder, unspecified	1 (0.27)	1 (0.004)	0 (0)	-
Mood disorder, with anxiety and depression	1 (0.27)	1 (0.004)	0 (0)	-
Narcissistic personality disorder	1 (0.27)	0 (0)	1 (0.009)	-
Panic disorder with agoraphobia	1 (0.27)	1 (0.004)	0 (0)	-
Paranoid personality disorder	1 (0.27)	1 (0.004)	0 (0)	-
Persistent depressive disorder	3 (0.80)	3 (0.011)	0 (0)	-
Schizophrenia	2 (0.54)	1 (0.004)	1 (0.009)	-
To consider schizophrenia, bipolar disorder	1 (0.27)	0 (0)	1 (0.009)	-

The discharge diagnosis at Table 4 was also collected as part of the data samples, and like the admitting diagnosis, a majority of the data sets are under the major depressive disorders. It was also noted to be significant under Fisher's exact test at 0.001499 (p<0.20).

**Table 4:** Discharge Diagnosis Data are presented as median and frequency (%). P-values<0.20 are significant ^f^Fisher's exact test

Discharge Diagnosis	Total (n=373)	Aggression		P-value
		Without (n=266)	With (n=107)	
Anxiety disorder, not otherwise specified	1 (0.27)	1 (0.004)	0 (0)	0.001499^f^
Bipolar 1 disorder	5 (1.34)	4 (0.015)	1 (0.009)	-
Bipolar 1 disorder with psychotic features	2 (0.54)	1 (0.004)	1 (0.009)	-
Bipolar 1 disorder with psychotic features, severe	1 (0.27)	1 (0.004)	0 (0)	-
Bipolar 1 disorder, most recent episode depressed	19 (5.09)	14 (0.053)	5 (0.047)	-
Bipolar 1 disorder, most recent episode depressed, in anxious distress	2 (0.54)	1 (0.004)	1 (0.009)	-
Bipolar 1 disorder, most recent episode depressed, with mixed and psychotic features	1 (0.27)	1 (0.004)	0 (0)	-
Bipolar 1 disorder, most recent episode depressed, with mixed features	3 (0.8)	1 (0.004)	2 (0.019)	-
Bipolar 1 disorder, most recent episode depressed, with psychotic features	3 (0.8)	2 (0.008)	1 (0.009)	-
Bipolar 1 disorder, most recent episode manic	55 (14.75)	24 (0.09)	31 (0.29)	-
Bipolar 1 disorder, most recent episode manic, with mixed features	1 (0.27)	1 (0.004)	0 (0)	-
Bipolar 1 disorder, most recent episode manic, with mood congruent psychotic features	1 (0.27)	1 (0.004)	0 (0)	-
Bipolar 1 disorder, most recent episode manic, with psychotic features	23 (6.17)	14 (0.053)	9 (0.084)	-
Bipolar 1 disorder, most recent episode mixed	11 (2.95)	9 (0.034)	2 (0.019)	-
Bipolar 1 disorder, most recent episode mixed, with psychotic features	2 (0.54)	1 (0.004)	1 (0.009)	-
Bipolar 1 disorder, most recent episode mixed, with psychotic features, in anxious distress	1 (0.27)	0 (0)	1 (0.009)	-
Bipolar 1 disorder, with mixed features, in anxious distress	2 (0.54)	2 (0.008)	0 (0)	-
Bipolar 2 disorder	7 (1.88)	6 (0.023)	1 (0.009)	-
Bipolar 2 disorder, in anxious distress	2 (0.54)	1 (0.004)	1 (0.009)	-
Bipolar 2 disorder, most recent episode depressed	10 (2.68)	9 (0.034)	1 (0.009)	-
Bipolar 2 disorder, most recent episode depressed, with mixed features	1 (0.27)	1 (0.004)	0 (0)	-
Bipolar 2 disorder, most recent episode hypomanic, with psychotic features	1 (0.27)	1 (0.004)	0 (0)	-
Bipolar 2 disorder, most recent episode mixed	2 (0.54)	2 (0.008)	0 (0)	-
Bipolar 2 disorder, most recent episode hypomanic, with mixed features	1 (0.27)	1 (0.004)	0 (0)	-
Bipolar disorder	30 (8.04)	15 (0.056)	15 (0.14)	-
Bipolar disorder with mixed and psychotic features	1 (0.27)	1 (0.004)	0 (0)	-
Bipolar disorder with psychotic features	2 (0.54)	1 (0.004)	1 (0.009)	-
Bipolar disorder, most recent episode depressed	8 (2.14)	7 (0.026)	1 (0.009)	-
Bipolar disorder, most recent episode depressed, with mixed features, in anxious distress	1 (0.27)	1 (0.004)	0 (0)	-
Bipolar disorder, most recent episode hypomanic	1 (0.27)	0 (0)	1 (0.009)	-
Bipolar disorder, most recent episode mixed	1 (0.27)	0 (0)	1 (0.009)	-
Bipolar disorder, most recent episode mixed, with psychotic features	1 (0.27)	1 (0.004)	0 (0)	-
Disruptive mood dysregulation disorder	1 (0.27)	0 (0)	1 (0.009)	-
Dysthymia	1 (0.27)	1 (0.004)	0 (0)	-
Major depressive disorder	103 (27.61)	83 (0.312)	20 (0.187)	-
Major depressive disorder with mixed features	1 (0.27)	1 (0.004)	0 (0)	-
Major depressive disorder with mixed features, in anxious distress	2 (0.54)	2 (0.008)	0 (0)	-
Major depressive disorder with psychotic features	27 (7.24)	22 (0.083)	5 (0.047)	-
Major depressive disorder with psychotic features, in anxious distress	1 (0.27)	1 (0.004)	0 (0)	-
Major depressive disorder, in anxious distress	25 (6.7)	23 (0.086)	2 (0.019)	-
Major depressive disorder, recurrent, with mixed and psychotic features	1 (0.27)	1 (0.004)	0 (0)	-
Mood disorder, unspecified	2 (0.54)	0 (0)	2 (0.019)	-
Other specified bipolar disorder	1 (0.27)	1 (0.004)	0 (0)	-
Panic disorder	1 (0.27)	1 (0.004)	0 (0)	-
Persistent depressive disorder	5 (1.34)	5 (0.019)	0 (0)	-
Anxiety disorder, not otherwise specified	1 (0.27)	1 (0.004)	0 (0)	-
Bipolar 1 disorder	5 (1.34)	4 (0.015)	1 (0.009)	-
Bipolar 1 disorder with psychotic features	2 (0.54)	1 (0.004)	1 (0.009)	-
Bipolar 1 disorder with psychotic features, severe	1 (0.27)	1 (0.004)	0 (0)	-
Bipolar 1 disorder, most recent episode depressed	19 (5.09)	14 (0.053)	5 (0.047)	-
Bipolar 1 disorder, most recent episode depressed, in anxious distress	2 (0.54)	1 (0.004)	1 (0.009)	-

Comorbid psychiatric disorders in Table 5 also show that most of the data sets showed no comorbidities (n=165). Alcohol abuse (n=10), anxiety disorder (n=8), and the non-accidental ingestion of multiple substances (n=8) were the top 3 of the classified comorbidities. In the classification of aggression, there is still a significantly increased number of data sets without a history of aggression (n=127). Under Fisher's exact test, the P-value garnered a value of 0.04498 and was considered significant (p<0.20).

**Table 5:** Comorbid Psychiatric Disorders Data are presented as median and frequency (%). P-values<0.20 are significant ^f^Fisher's exact test

Comorbid Psychiatric Disorders	Total (n=373)	Aggression		P-value
		Without (n=266)	With (n=107)	
Attention deficit hyperactivity disorder	1 (0.27)	1 (0.004)	0 (0)	0.04498^f^
Attention deficit hyperactivity disorder, polysubstance use disorder	1 (0.27)	1 (0.004)	0 (0)	-
Adjustment disorder	1 (0.27)	1 (0.004)	0 (0)	-
Alcohol abuse	10 (2.68)	5 (0.019)	5 (0.047)	-
Alcohol abuse, pathological gambling	1 (0.27)	0 (0)	1 (0.009)	-
Alcohol and benzodiazepine abuse	1 (0.27)	0 (0)	1 (0.009)	-
Alcohol and benzodiazepine abuse, non-accidental ingestion of diphenhydramine	1 (0.27)	1 (0.004)	0 (0)	-
Alcohol and cannabinoid abuse	1 (0.27)	1 (0.004)	0 (0)	-
Alcohol and cannabinoid abuse	1 (0.27)	0 (0)	1 (0.009)	-
Alcohol and methamphetamine abuse	1 (0.27)	1 (0.004)	0 (0)	-
Alcohol and polysubstance use disorder	2 (0.54)	0 (0)	2 (0.019)	-
Alcohol and substance dependency	1 (0.27)	0 (0)	1 (0.009)	-
Antisocial personality disorder	1 (0.27)	0 (0)	1 (0.009)	-
Anxiety disorder	8 (2.14)	7 (0.026)	1 (0.009)	-
Anxiety disorder, insomnia disorder	1 (0.27)	1 (0.004)	0 (0)	-
Anxiety disorder, methamphetamine dependence	1 (0.27)	1 (0.004)	0 (0)	-
Anxiety disorder, non-accidental ingestion of alprazolam	1 (0.27)	1 (0.004)	0 (0)	-
Autism spectrum disorder	2 (0.54)	2 (0.008)	0 (0)	-
Autism spectrum disorder, attention deficit hyperactivity disorder	1 (0.27)	0 (0)	1 (0.009)	-
Autism spectrum disorder, without intellectual impairment	1 (0.27)	1 (0.004)	0 (0)	-
Benzodiazepine dependence	1 (0.27)	1 (0.004)	0 (0)	-
Borderline personality disorder	7 (1.88)	3 (0.011)	4 (0.037)	-
Bulimia nervosa	1 (0.27)	1 (0.004)	0 (0)	-
Cannabinoid abuse	7 (1.88)	4 (0.015)	3 (0.028)	-
Cannabinoid and lysergic acid diethylamide abuse	1 (0.27)	0 (0)	1 (0.009)	-
Cannabinoid and methamphetamine abuse	1 (0.27)	0 (0)	1 (0.009)	-
Cluster B personality disorder	1 (0.27)	1 (0.004)	0 (0)	-
Cocaine abuse	2 (0.54)	1 (0.004)	1 (0.009)	-
Cocaine and cannabinoid abuse	1 (0.27)	0 (0)	1 (0.009)	-
Dissociative identity disorder	1 (0.27)	0 (0)	1 (0.009)	-
Gambling addiction	1 (0.27)	1 (0.004)	0 (0)	-
Gender dysphoria	1 (0.27)	1 (0.004)	0 (0)	-
Generalized anxiety disorder	1 (0.27)	1 (0.004)	0 (0)	-
Generalized anxiety disorder, non-accidental ingestion of multiple substances	1 (0.27)	1 (0.004)	0 (0)	-
Ingestion of 14 tablets of quetiapine	1 (0.27)	1 (0.004)	0 (0)	-
Ingestion of loratadine and loperamide	1 (0.27)	1 (0.004)	0 (0)	-
Insomnia disorder	1 (0.27)	1 (0.004)	0 (0)	-
Intellectual disability	1 (0.27)	0 (0)	1 (0.009)	-
Major neurocognitive disorder, delirium secondary to urinary tract infection	7 (1.88)	1 (0.004)	0 (0)	-
Methamphetamine abuse	6 (1.61)	5 (0.019)	2 (0.019)	-
Not applicable	1 (0.27)	2 (0.008)	4 (0.037)	-
Nitrous oxide abuse	1 (0.27)	0 (0)	1 (0.009)	-
Non-accidental ingestion	1 (0.27)	1 (0.004)	0 (0)	-
Non-accidental ingestion of alprazolam	1 (0.27)	1 (0.004)	0 (0)	-
Non-accidental ingestion of amitriptyline	1 (0.27)	1 (0.004)	0 (0)	-
Non-accidental ingestion of carbamate	1 (0.27)	1 (0.004)	0 (0)	-
Non-accidental ingestion of estazolam	1 (0.27)	0 (0)	1 (0.009)	-
Non-accidental ingestion of multiple substances	8 (2.14)	7 (0.026)	1 (0.009)	-
Non-accidental ingestion of paracetamol	1 (0.27)	1 (0.004)	0 (0)	-
None	165 (44.24)	127 (0.477)	38 (0.355)	-
Obsessive-compulsive disorder	4 (0.8)	3 (0.011)	1 (0.009)	-

Table 6 shows that mood states such as manic, mixed, and anxious significantly increase the aggressive behavior of the patients using logistic regression. Specifically, individuals in manic, mixed, or anxious states can expect their outcomes to be 5-6 times greater than those who are depressed. These values were also noted to be significant (p<0.20) with manic (p=1.59435e^-09^), mixed (p=2.60205e^-04^), and anxious (p=2.880880e^-07^).

**Table 6:** Logistic Regression Table on How Mood State or Episode Is Associated With Aggressive Behavior P-value<0.20 is significant. Likelihood ratio test: 53.2603. Wald's ratio rest: 84.6619

Mood State	Odds Ratio (OR)	95% CI (Lower, Upper)	P-value
Depression	0.165	-2.209, -1.427	0.00
Manic	5.98	1.1951, 2.3708	1.59435e^-09^
Mixed	5.57	0.8177, 2.615158	2.60205e^-04^
Anxious	5.46	1.0536, 2.3499	2.880880e^-07^
Unspecified	2.59	-1.4062, 2.7996	0.0371
Euthymic	0.86	-5.0567, 2.2248	0.9225

## Discussion

Starting with the demographics as shown in Table 1, the data confirmed known facts such as female gender having predominance in the sample, especially with major depressive disorder being considered in this study [2,4,10], and a study [1] showed a sample population that was dominated by male participants. The data summary indicated that among the demographic variables, civil status shows a statistically significant association with the presence or absence of aggression (p=0.004998). It validates the idea that loneliness has an effect on cultivating aggression [10,11] and that gender has no significant effect on the prevalence of aggression (p=0.1712) [4,12]. The study also shows that age (p=0.0489), employment (p=0.508), and religious affiliation (p=0.8987) do not have a significant association with the presence or absence of aggression, which is distinctly in contrast to a study wherein non-employment was concluded to be a protective factor [13].

In this inquiry regarding the clinical determinants as shown in Table 2, the mood state during admission (p=2.798e^-10^), the patients' mood symptoms at the time of admission (p=1.127e^-12^), the type of aggression (p=2.2e^-16^), and the total number of symptoms for depression (p=3.462e^-10^) and mania (p=3.445e^-11^) demonstrate significant associations with the presence or absence of aggression. However, an unexpected determinant that was noted to have no significant association with the absence or presence of aggression was the suicide attempts. In some studies, suicide history was usually reported as a risk factor [13,14]. Another study suggests that the neurobiological connection of aggression and impulsivity with suicide risk is seen through magnetoencephalography. It reports that there is reduced activity and connectivity in the regions of the brain concerned with sensory and emotional regulation in patients who recently had a suicidal crisis [15]. Another determinant that was known to be a factor in considering aggression was the number of mood episodes (p=0.8078), which yielded no significant association with the absence or presence of aggression in this study. This is contradictory to the idea that having a recent mood episode wherein a patient experienced an affective change, it is more likely for the patient to have another bout of emotional dysregulation resulting in aggression [14,16].

This study shows that admitting diagnosis (p=0.004498) and discharge diagnosis (p=0.001499) have significant associations with the presence or absence of aggression. These validate that having a diagnosis under mood disorders has an effect on the presence of aggression as a symptom presented by a patient [10,12]. For the comorbid psychiatric diagnosis in Table 5 (p=0.04498), analysis also noted a significant association with the presence or absence of aggression. It has been reported that certain psychiatric disorders are known to increase the risk of aggression, and this holds true with the results of these inquiries, especially with the comorbid psychiatric disorders [12]. As evident in most of the tables, the sample notably had more of the data sets classified as having no aggression.

As shown in Table 6, the manic, mixed, and anxious mood states had 5-6 times more chances to present aggression. However, the mood states of unspecified mood and euthymic mood do not show significant differences from the depressed state, implying that these mood conditions are associated with outcomes similar to those seen in depression. The likelihood ratio and Wald tests indicate a strong overall model fit, suggesting that mood states collectively are associated with the aggressive behavior of the patient. Although it cannot be said that depressive states have increased expected outcomes with those on euthymic mood states, other studies still say that the presence of depression can cause general aggression, with self-aggression a more likely result [17,18]. Also, in a recent study, wherein aggression, hostility, and anger were differentiated through subscales with a questionnaire, only anger and hostility were noted to be correlated with depressive state [10].

In a manic and mixed state, patients would usually characterize irritability as part of the symptom constellation. Studies show that irritability in this state experienced by the patient was associated with aggression [15,18,19]. In our study, an anxious state, similar to manic and mixed states, has an increased expected outcome of aggression, which is the same in some studies, but they also correlated it to paranoia experienced by patients in this kind of state, making paranoia a factor to consider in treatment and management [19,20].

The study tried to prove whether aggression toward oneself has a role in predicting overall aggression; however, it failed to show any statistical correlation due to its limitations. An enhancement on the design of the study to explore the dynamics and spectrum of aggression in patients with previous suicidal attempts may be needed. Prospective design showing instances of aggression toward oneself and toward others might be considered. This may warrant further investigation that could shed a better light on this information. Another limitation was the sample; this study concentrated on admitted patients and 373 data sets gathered. The conclusions may not have a direct applicability to the general population, as the study did not include data sets from patients who were managed from an outpatient setting. Considering that both inpatient and outpatient may have a much more representative result, the number of data sets, although computed to be significant, could be increased to make the evidence much more credible.

## Conclusions

In summary, it can be safely concluded that the factors with significant association to the absence and presence of aggression (civil status, mood state during admission, patients' mood symptoms at the time of admission, the type of aggression, the total number of symptoms for depression and mania, admitting diagnosis, discharge diagnosis, and comorbid psychiatric disorders) are also known considerations for assessing the probability of aggression, either toward others or of suicidal nature. Clinically, these validate some of the known parameters in predicting future aggression either toward oneself or others, which can be helpful in managing patients suffering from mood disorders.

Furthermore, the recent mood state was shown to have a positive association in terms of predicting the probability of aggression, especially manic, anxious, and mixed states. It might be a good cause for inquiry into how anxiety affects the presentation of mood disorders and how it can be properly managed. One of the primary purposes of this inquiry was to examine whether there is a change in terms of predictors of aggression if the concept of aggression includes any violence committed toward oneself. Some of the predictors held true, but contradictory evidence from current literature was also gathered.

## Appendices

Table 7 shows the data collection tool.

**Table 7:** Data Collection Tool F, female; M, male; S, single; M, married; Se, separated; Dx, diagnosis

-	-	0-F; 1-M	1-S; 2-M; 3-Se; 4-Widowed/Widower	1-Catholic; 0-Non-Catholic	-	-	-	0-Depressed; 1-Manic; 2-Anxious; 3-Mixed; 4-Unspecified; 5-Euthymic	-
Control number	Age	Gender	Civil status	Religious affiliation	Employment	Admitting Dx	Discharge Dx	Mood state at the time of admission	Number of episodes/year
-	-	-	-	-	-	-	-	-	-
